# High pressure effects revisited for the cuprate superconductor family with highest critical temperature

**DOI:** 10.1038/ncomms9990

**Published:** 2015-12-01

**Authors:** Ayako Yamamoto, Nao Takeshita, Chieko Terakura, Yoshinori Tokura

**Affiliations:** 1 Strong Correlation Physics Division, RIKEN Center for Emergent Matter Science (CEMS), Wako 351-0198, Japan; 2 Electronics and Photonics Research Institute, National Institute of Advanced Industrial Science and Technology (AIST), Tsukuba 305-8568, Japan; 3 Department of Applied Physics, The University of Tokyo, Tokyo 113-8656, Japan

## Abstract

How to enhance the superconducting critical temperature (*T*_c_) has been a primary issue since the discovery of superconductivity. The highest *T*_c_ reported so far is 166 K in HgBa_2_Ca_2_Cu_3_O_8+δ_ (Hg1223) at high pressure of 23 GPa, as determined with the reduction onset, but not zero, of resistivity. To clarify the possible condition of the real maximum *T*_c_, it is worth revisiting the effects of pressure on *T*_c_ in the highest *T*_c_ family. Here we report a systematic study of the pressure dependence of *T*_c_ in HgBa_2_CaCu_2_O_6+δ_ (Hg1212) and Hg1223 with the doping level from underdoped to overdoped. The *T*_c_ with zero resistivity is probed with a cubic-anvil-type apparatus that can produce hydrostatic pressures. Variation, not only increase but also decrease, of *T*_c_ in Hg1212 and Hg1223 with pressure strongly depends on the initial doping levels. In particular, we confirm a maximum *T*_c_ of 153 K at 22 GPa in slightly underdoped Hg1223.

## Abstract (web summary)

Superconductivity, zero-resistance electrical transport, exists up to temperatures above 150 K in mercury-based cuprates under pressure. Here, the authors revisit the pressure dependence of the superconducting transition temperature in these materials and find a strong dependence on initial doping.

## Graphical abstract

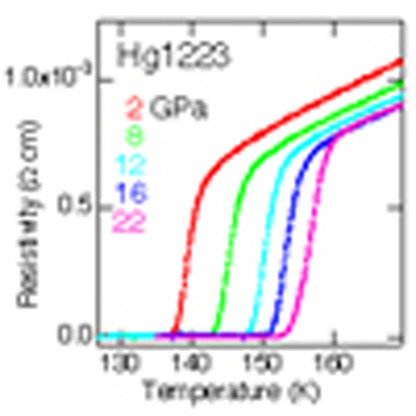

Knowledge of the effects of pressure on superconducting critical temperature (*T*_c_) is of paramount importance for understanding the mechanism of superconductivity, as exemplified by the latest report on the pressure-induced above-200 K superconductivity^1^. In the conventional superconductors such as MgB_2_, *T*_c_ decreases with increasing pressure reflecting the decrease of density-of-states at the Fermi level^2^, whereas in some of the unconventional superconductors such as FeSe, *T*_c_ increases with increasing pressure^3^ at least within a certain range of pressure. In the most of high-*T*_c_ superconductors (HTSCs) with CuO_2_ layers, *T*_c_ increases with pressure in a manner strongly depending on the composition and doping level^4^.

Mercury-based HTSCs denoted as the general formula HgBa_2_Ca_*n*−1_Cu_*n*_O_2*n*+2+δ_ (*n*=1, 2, 3, 4,...) show very high values of *T*_c_ already at ambient pressure (AP): 95, 127, 134, and 130 K for *n*=1, 2, 3, and 4, respectively^5^,^6^,^7^. Studies of pressure effects on *T*_c_ were started just after the discovery of this homologous series, and the maximum values of *T*_c_ for HgBa_2_CaCu_2_O_6+δ_ (Hg1212) and HgBa_2_Ca_2_Cu_3_O_8+δ_ (Hg1223) were reported to be 154 K (ref. 8) and 166 K (ref. 9) at ∼25 GPa, respectively. Gao *et al.*^8^ argued that a universal upward shift in *T*_c_ was a common feature for all Hg 12(*n*−1)*n*. Although these values of *T*_c_ are commonly accepted, we believe they should be reinvestigated to better understand the effects of high pressures in the light of advances in materials synthesis and high pressure technique; these values of *T*_c_ were determined from the so-called onset temperature, at which resistivity begins to deviate at a steeper rate from those at higher temperatures with decrease of temperature, whereas the observation of zero resistivity, which literally defines superconductivity, was not attained. Furthermore, in previous studies the pressure above 10 GPa was generated with a diamond-anvil cell^8^,^9^, which does not necessarily ensure the generation of pressures of excellent hydrostatic nature. For a precise study on effects of pressure on zero resistance *T*_c_, high-quality hydrostatic pressures are required.

To address these issues, we have reinvestigated the effects of pressure on *T*_c_ in Hg-HTSCs using high-pressure-synthesized dense polycrystalline samples and nearly hydrostatic pressures. Recently, we briefly reported that *T*_c_ above 150 K could be achieved with zero resistivity in pressurized Hg1223 (ref. 10). In the present work, we study the pressure dependence of *T*_c_ in both Hg1212 and Hg1223 over a wide range of doping level; for Hg1212 a large and critical doping dependence of the pressure effect on *T*_c_ is revealed, while for Hg1223 the *T*_c_ with zero resistivity increases monotonously with pressure and reaches a maximal value of 153 K at 22 GPa.

## Results

### Doping dependence of resistivity at ambient pressure

**Figure 1:**
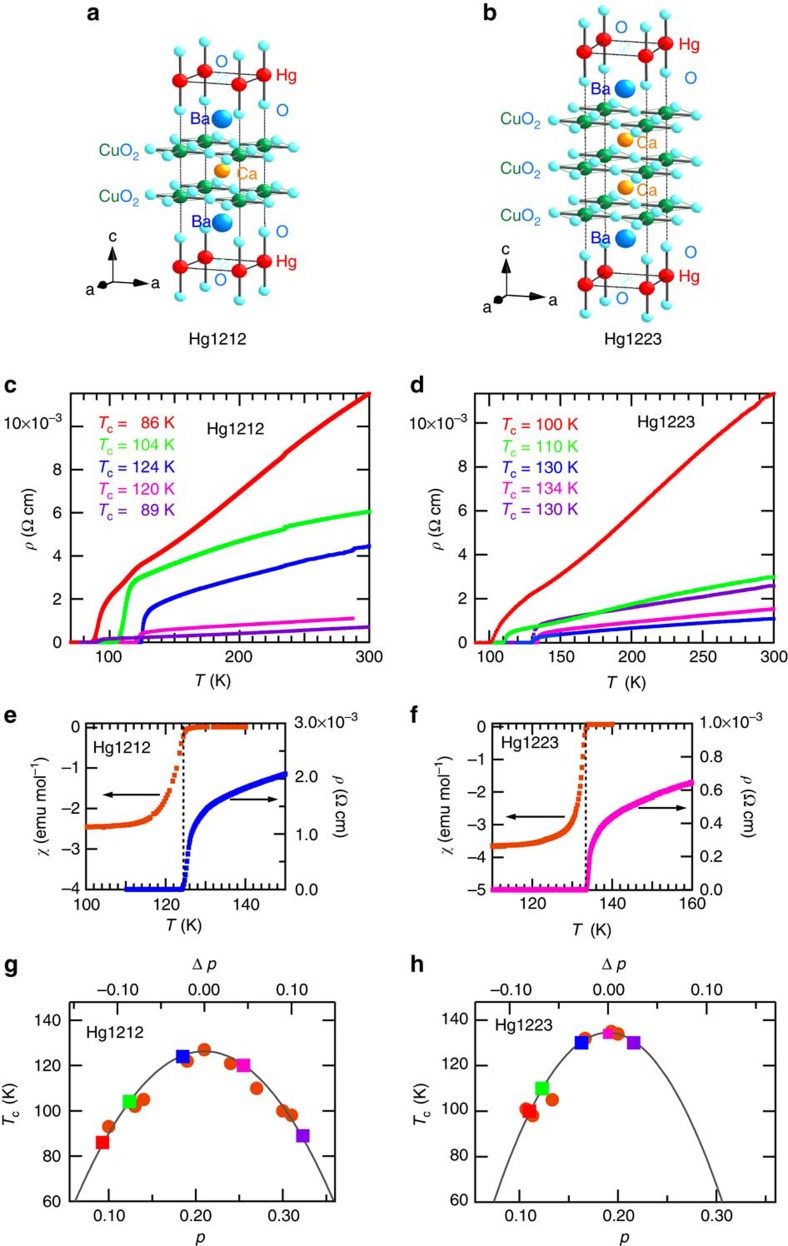
Characterization of Hg1212 and Hg1223. (**a**,**b**) Crystal structure models of Hg1212 (**a**) and Hg1223 (**b**). Red, blue, yellow, green and light blue spheres correspond to Hg, Ba, Ca, Cu and O, respectively. Hashed circle indicates partially occupied oxygen. (**c**,**d**) Temperature dependence of resistivity (*ρ*–*T*) of Hg1212 (**c**) and Hg1223 (**d**) with various *T*_c_. Colour of the curve corresponds to the text colour of *T*_c_. (**e**,**f**) Correlation between *ρ*–*T* and temperature dependence of dc-susceptibility (*χ*–*T*) curves exemplified for Hg1212 (*T*_c_=124 K) and Hg1223 (*T*_c_=134 K). (**g**,**h**) Estimation of hole concentration (*p*) for Hg1212 (**g**) and Hg1223 (**h**) from a fitting curve (line) using the data in ref. 13 (solid circle). Squared symbols are data of present study. Colour of the symbol corresponds to the colour of the resistivity in panels of (**c**,**d**). Δ*p* stands for the value measured from the optimal doping value at ambient pressure; namely, Δ*p*<0 for underdoped and Δ*p*>0 for overdoped samples.

Figure 1a,b show the crystal structures of Hg1212 and Hg1223. These structures offer advantages for investigation of pressure effects. One is the flatness of conductive CuO_2_ layer^11^, which would be an important factor for the observed highest *T*_c_ in HTSCs. This is attributed to coordination of the CuO_2_ layer with an elongated Cu–O pyramid, which has a long distance (2.7208(3) Å) between the apical-O (O_a_) and Cu owing to a short covalent bond (1.972(3) Å) between Hg and O_a_ (ref. 11). Another advantage is that the so-called charge-reservoir block-layer (HgO_δ_) is located away from the CuO_2_ layer, therefore the extra oxygen least affect local distortion or least cause random potential in CuO_2_ layers. Comparison of high pressure effects in Hg1212 and Hg1223 are also interesting, because Hg1212 is composed of only the equivalent two outer CuO_2_ layers with an apex-elongated pyramid, while Hg1223 has extra one inner layer of CuO_2_ square plane sandwiched by the two outer CuO_2_ layers.

The temperature (*T*) dependences of electrical resistivity (*ρ*) in Hg1212 and Hg1223 at AP are shown in Fig. 1c,d. The doping variations in *ρ*–*T* curves exhibit common features of in-plane resistivity of HTSC cuprates^12^. The insets show the dc-susceptibility and resistivity of nearly optimally doped samples and confirm that the diamagnetic (Meissner effect) signal starts at the temperature of zero resistivity. This defines the temperature of the superconducting transition, and in the following we adopt such a zero-resistivity temperature as *T*_c_.

Hole doping level of each sample was estimated from the experimentally established relationship between hole doping and *T*_c_ reported in ref. 13, as shown in Fig. 1e,f. Values of *T*_c_ were determined from the temperature at which the diamagnetic signal begins to appear. We set Δ*p*=*p*−*p*_max_, where *p*_max_ gives the maximum *T*_c_ at AP in the system; by definition, Δ*p*<0 for the underdoped samples and Δ*p*>0 for the overdoped samples. Note that the variation in hole concentration of Hg1212 is wider than that of Hg1223. It was difficult to obtain an enough overdoped sample for Hg1223, even after it was annealed under high-pressure oxygen.

### Doping dependence of resistivity at high pressures

**Figure 2:**
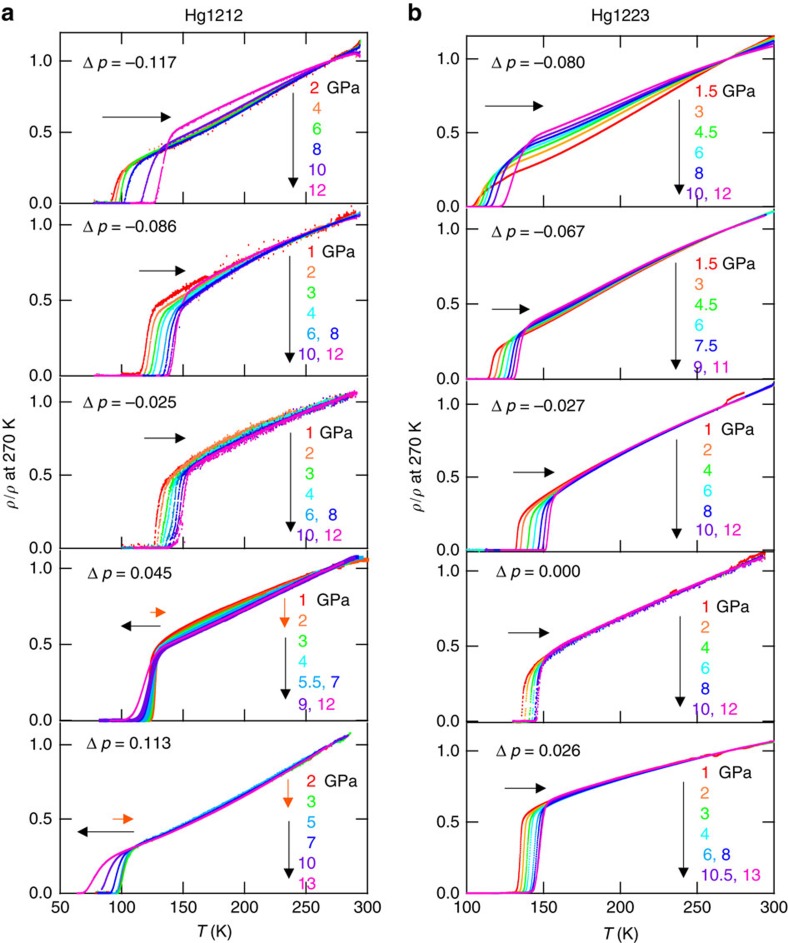
Temperature dependence of electrical resistivity of Hg1212 and Hg1223 at high pressures. (**a**,**b**) Temperature dependence of electrical resistivity normalized at 270 K for Hg1212 (**a**) and Hg1223 (**b**) samples with wide doing levels indicated by Δ*p*. Colour of the curve corresponds to the text colour of pressure.

The *ρ*–*T* curves of Hg1212 and Hg1223 up to 11–13 GPa are shown in Fig. 2. To facilitate visualization, the resistivities are normalized by the respective values at 270 K, while the absolute values of resistivity reduce by 30–70% from AP to 11–13 GPa. In both Hg1212 and Hg1223, the *ρ*–*T* curve in the underdoped (Δ*p*<0 ) region gradually changes from an upward convex to a straight line, though the degree of the change depends on the initial Δ*p* values. The superconducting transition itself remains sharp, even at high pressure over 10 GPa. In the positive Δ*p* (overdoped) region for Hg1212, however, the *ρ*–*T* curve is convex downward typical of a normal metal, while the superconducting transition becomes broader with increasing pressure. The origin of the broadened transition under pressures in the overdoped region for Hg1212 is not clear, but such a conspicuous broadening in the resistive transition is also seen for the underdoped region under higher pressures (for example, >15 GPa) where *T*_c_ turns to decrease with further application of pressure. (We come back to this problem with speculative discussion.) Note that the *T*_c_'s for the Hg1212 samples with Δ*p*=0.045 (d) and Δ*p*=0.113 (e) show non-monotonous pressure dependence, namely at first increase and then decrease with increasing pressure. The reason for the rapid increase in *T*_c_ at relatively low pressure may be ascribed to a pressure-induced structural change, such that the apical oxygen rapidly approaches the CuO_2_ layer^14^. Details pressure dependences of *T*_c_ and d*T*_c_/d*P* in Hg1212 and Hg1223 are shown in the Supplementary Fig. 1.

**Figure 3:**
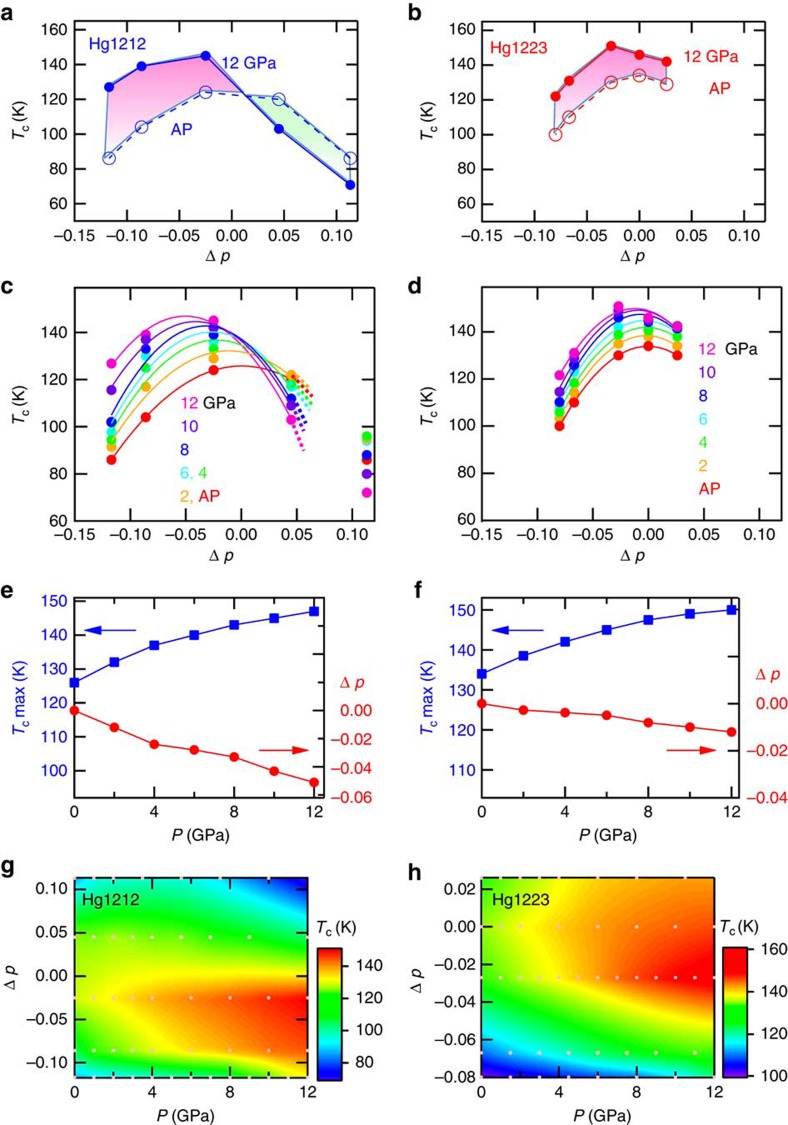
Effects of pressure on *T*_c_ of Hg1212 and 1223. (**a**,**b**) Hole doping level (Δ*p*) dependence of *T*_c_ in Hg1212 (**a**) and Hg1223 (**b**) at ambient pressure (filled circle and solid line) and 12 GPa (open circle and dashed line ). Areas filled with pink and green show *T*_c_ -increased and—decreased, respectively, with pressure. (**c**,**d**) Measured and interpolated values *of T*_c_ as a function of Δ*p* for Hg1212 (**c**) and Hg1223 (**d**) at high pressures. The parabolic solid lines show the fitting for the constant-pressure *T*_c_ versus Δ*p* curves. Colour of the line and circle corresponds to the text colour of the pressure. (**e**,**f**) Pressure (*P*) dependence of *T*_c_ maximum (blue square) and Δ*p* (red circle) that provides *T*_c_ maximum for Hg1212 (**e**) and Hg1223 (**f**). These values are estimated by the fitting curves of panel (**c**,**d**). (**g**,**h**) *T*_c_ contour maps on pressure doping level (*P*–Δ*p*) diagram in Hg1212 (**e**) and Hg1223 (**f**). A colour bar shows *T*_c_ value in Kelvin. The dots in pale pink correspond to the measured data points.

Figure 3 shows the Δ*p* dependence of *T*_c_ in Hg1212 and Hg1223. Figure 3a exemplifies the variation of *T*_c_ between at AP and 12 GPa in Hg1212. The values of *T*_c_ in the samples with negative Δ*p* are increased by more than 30 K with application of pressure up to 12 GPa; notably in the most underdoped (Δ*p*=−0.113) sample the *T*_c_ is increased as much as by 41 K. In contrast, the samples with positive Δ*p* show the pressure-induced reduction of *T*_c_. Such a clear reduction of *T*_c_ with pressure has rarely been reported in HTSC cuprates except for overdoped Tl_2_Ba_2_CuO_6+δ_ (Tl2201) (ref. 15). In Hg1223, on the other hand, *T*_c_ is simply increased by about 20 K from AP up to 12 GPa in each sample shown in Fig. 3b irrespective of positive and negative value of Δ*p*, while the pressure effect on *T*_c_ appears smaller as compared with those for Hg1212 (cf. Supplementary Fig. 1).

Figure 3c,d shows *T*_c_ values as a function of Δ*p* at various pressures (at interval of 2 GPa) for Hg1212 and Hg1223, respectively. The parabolic solid lines represent the fitting curves connecting the constant-pressure *T*_c_s. This parabolic fitting to the Δ*p* versus *T*_c_ dome appears to fail to cover the overdoped region, for example, Δ*p*=0.113 for Hg1212, yet can work well for the extrapolation to determine the maximal *T*_c_ and the corresponding Δ*p* value. From these curves, we obtain the realizable maximal *T*_c_ and the corresponding Δ*p* at respective pressure values, as shown in Fig. 3e,f. Those plots show a clear tendency that the maximal *T*_c_ is attained at lower Δ*p* and higher pressures. We can also see such correlation in the *T*_c_ contour maps on the doping–pressure diagram in Fig. 3g,h. All these analyses suggest that the maximal *T*_c_ under pressures does not always occur at the optimal doping level at AP; the optimal doping giving rise to the highest *T*_c_ does depend on the pressure value. The possible highest *T*_c_ is expected at lower doping levels and higher pressures. In practice, however, it was difficult to generate a hydrostatic pressure above 13 GPa in the present pressure load cell with an anvil size of 4 mm side.

### Resistivity at high pressure over 20 GPa

**Figure 4:**
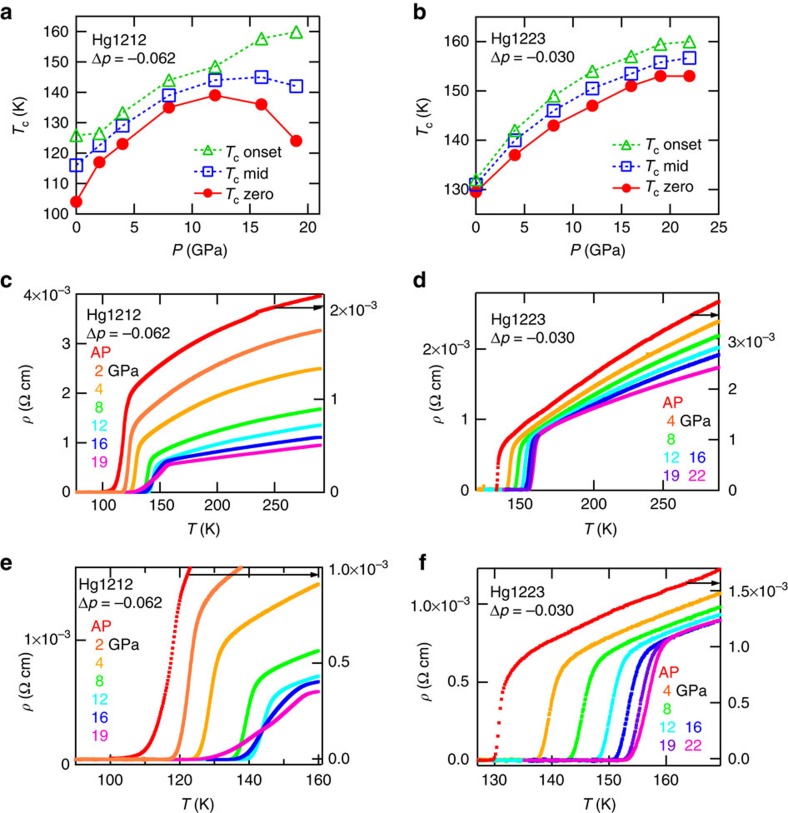
Effects of higher pressure on superconductivity in Hg1212 and Hg1223. (**a**,**b**) Pressure (*P*) dependence of *T*_c zero_ (zero resistivity), *T*_c mid_ (a peak position of d*ρ/*d*T* in superconducting transition), and *T*_c onset_ (the resistivity to start to decrease toward zero, as defined by a rising edge in dρ/d*T*) for Hg1212 (**a**) and Hg1223 (**b**). (**c**,**d**) Temperature dependence of electrical resistivity (*ρ*–*T*) for Hg1212 (**c**) and Hg1223 (**d**) Colour of curve corresponds to the text colour of pressure. (**e**,**f**) Zoom-up figures of (**c**–**f**).

To realize a higher *T*_c_, we tried to generate higher pressures using a newly developed anvil of 3 mm side. This enabled us to achieve a nearly hydrostatic pressure up to 22 GPa, which, to the best of our knowledge, would be the highest pressure attained with a cubic-anvil press for resistivity measurements. The detailed procedure and specification for the higher pressure measurement will be reported elsewhere. In this work, we applied pressures of up to 19–22 GPa on four samples; Hg1212 with Δ*p*=−0.062, and Hg1223 with Δ*p*=−0.030, −0.052 and −0.095 to explore the possible highest *T*_c_s (zero resistance) under realistic hydrostatic pressures. The hole doping levels of these samples should be nearly optimal to enhance *T*_c_ at around 20 GPa as expected from the results shown in Fig. 3c–h. Effects of pressure on *T*_c_ for the Hg1212 and the Hg1223 samples are shown in Fig. 4a,b together with *T*_c mid_ (peak temperature in d*ρ*/d*P* curve) and *T*_c onset_ (temperature for resistivity to start declining towards zero resistivity). Temperature dependences of resistivity are shown in Fig. 4c,d. Those for other samples of Hg1223 are shown in Supplementary Figs 2 and 3.

For Hg1212, *T*_c_ increases up to 12 GPa with the maximum *T*_c_ of 140 K; above 12 GPa, *T*_c_ (as defined by zero resistance) decreases although the onset temperature for the resistivity reduction, *T*_c onset_, continues to rise. The *ρ*–*T* curve is slightly convex upward and the transition width suddenly becomes broad above 12 GPa, as observed in the overdoped samples of Hg1212 (see Fig. 2d,e). In Hg1223, *T*_c_ increases up to 22 GPa with the maximum *T*_c_ of 153 K. Above 19 GPa, *T*_c_ appears to saturate, although *T*_c mid_ and *T*_c onset_ continue to increase, as observed in the former study^10^. Thus, there still remains the possibility of further increasing *T*_c_ by applying higher pressure to samples with even lower Δ*p*.

## Discussion

In the following, we discuss the possible origins of the observed conspicuous pressure effects on *T*_c_. At first, we should note the highly non-monotonous pressure dependence of *T*_c_ in heavily underdoped samples (for example, Hg1212 with Δ*p*=−0.11 and Hg1223 with Δ*p*=−0.08) as shown in Fig. 3g,h and more clearly seen in Supplementary Fig. 3; at the first stage of applying pressure, *T*_c_ does not rise so much, but above 7–8 GPa suddenly increases with the high rate, and then the rate becomes moderate again. This newly found behaviour in *T*_c_–*P* curve for the underdoped region might trace the *T*_c_ plateau and jump in the *T*_c_–*p* curve, indicative of the charge order or stripe order that is characteristic of the low-doped CuO_2_ sheet with the reduced *T*_c_ (ref. 16); such a charge order instability may be removed by pressure above some critical value, for example, 7–8 GPa in the present case.

Next, we focus on the difference between the effects for Hg1212 and Hg1223 as exemplified in Fig. 3a,b. The Hg1212 shows clear increase and decrease in *T*_c_ with application of high pressure in the underdoped and overdoped regime, respectively, while the Hg1223 always shows the increase. One possible reason for this different doping dependence is the fact that only the narrower doping range can be prepared (or the enough overdoped state is difficult to form) for Hg1223 as compared with Hg1212. Another more essential reason is the presence of nonequivalent, that is, inner and outer, CuO_2_ layers involved in Hg1223. A former NMR study suggests that the copper valence of the inner layer of HgBa_2_Ca_4_Cu_5_O_12+δ_ (Hg1245) and TlBa_2_Ca_3_Cu_4_O_10+δ_ (Tl1234) are lower than those of the outer layer^17^. Redistribution of hole carriers is likely induced among the inner and outer CuO_2_ sheets in Hg1223 by applying pressure, which makes the mechanism of pressure-induced *T*_c_-change in Hg1223 more elusive than in Hg1212 where the hole-dopable unit is restricted to the crystallographically equivalent CuO_2_ sheets with the Cu–O pyramidal coordination. Thus the steeper increase (decrease) of *T*_c_ with pressure in an underdoped (overdoped) regime should be viewed as the intrinsic feature of the CuO_2_ sheets.

From the analyses shown in Fig. 3, in particular for Hg1212, we obtained the result that *T*_c_ maximum can be realized when starting from an underdoped sample rather than starting from an optimally doped sample. The initial doping level that provides the maximum *T*_c_ depends on the pressure value, as indicated in Fig. 3e,f. The steep increase and then decrease of *T*_c_ with pressure as seen in the underdoped (Δ*p*=–0.062) Hg1212 (Fig. 4a) indicates the empirical tendency that application of hydrostatic pressure effectively drives the system toward the overdoping regime; in this particular compound, for example, *T*_c_ (zero resistance) tends to decrease as the pressure exceeds 12 GPa, as if the compound were placed in the overdoped state (see Fig. 4a). Furthermore, being characteristic of the overdoped Hg1212 samples (Fig. 2e), the superconducting resistive transition becomes broadened in temperature, as manifested by a large discrepancy between *T*_c onset_ and *T*_c_ (zero resistance) in the *P*=16 and 19 GPa data of Fig. 4a. In particular, the high pressure (19 GPa) value of *T*_c onset_ for Hg1212 reaches 160 K as high as in the case of Hg1223 (Fig. 4b). This is the feature commonly observed in previous high-pressure studies^8^,^9^ on this highest *T*_c_ superconductor family. The origin of this broadening of the resistive transition with higher *T*_c onset_ but lower *T*_c_ (zero resistance) in the effectively pressure-induced overdoping regime is still not clear; it is hardly ascribable to the pressure inhomogeneity at least in the present experiment, since the superconducting transition under around 20 GPa for Hg1223 (see Fig. 4d) remains as sharp as at lower pressures. We speculate that the pressure-driven overdoped state may show the tendency of some intrinsic electronic phase separation into the higher *T*_c_ (>160 K) filaments or embryos and the lower *T*_c_ (<160 K) state above 20 GPa; however, this issue remains as an important subject of future study.

## Methods

### Sample preparation at high pressure

The polycrystalline samples of Hg1212 and Hg1223 were synthesized with a cubic–anvil-type high-pressure apparatus (180 ton press, TRY Eng. Co.). Mixtures of HgO, BaO_2_ CaO, and CuO/Cu, or mixtures of HgO and the precursor (prepared from BaCO_3_, CaO and CuO at 950 °C in a flowing high-purity oxygen gas) were placed into a cylindrical gold capsule and sintered for 10–30 min at 805–850 °C under a pressure of 2 GPa. To control the hole doping level, we have optimized the starting material, sintering condition, and annealing condition for each sample. Details are described in Supplementary Table 1.

### Sample characterization

The optimized nominal compositions to obtain single-phase materials were Hg_0.85_Ba_2_CaCu_2.15_O_6+δ_ for Hg1212 and Hg_0.75_Ba_2_Ca_2_Cu_3.25_O_8+δ_ for Hg1223. The analysed compositions determined by a scanning electron microscope (JSM-6701 F, JEOL) equipped with energy dispersive X-ray analyzer (XFlash detector 5030, Bruker AXS) were Hg:Ba:Ca:Cu=0.77:1.99:0.99:2.25 and 0.81:1.96:2.17:3.07, respectively. The powder X-ray diffraction patterns (RINT, Rigaku) confirmed the nearly single phase of Hg1212 and Hg1223. The scanning electron microscope images indicate that they are much denser than the samples prepared with the conventional silica-tube encapsulation method. The bulk superconductivity at ambient pressure was confirmed by dc*-*susceptibility measured with the field cooling procedure at 20 Oe (MPMS, Quantum Design) as well as by electrical resistivity (PPMS, Quantum Design).

### Resistivity measurement at high pressure

The electrical resistivity at high pressure was measured using a cubic-anvil-type high-pressure apparatus with low-temperature cryostat (200 ton press, Rockgate Co.,). The samples with the four gold wires were placed in Teflon capsule with grease (Apiezon N) as a pressure-transmitting medium, and put in the gasket of MgO. To produce higher pressure, we use anvil tops with the sintered diamond. The anvils with the size of 4 × 4 mm^2^ (sample size: 1.2 × 0.6 × 0.4 mm^3^) and of 3 × 3 mm^2^ (sample size: 0.8 × 0.4 × 0.3 mm^3^) were used for the experiments up to 13 and 22 GPa, respectively. The sample temperature was determined with the Cernox resistance thermosensor on one of the anvil top.

## Additional information

**How to cite this article:** Yamamoto, A. *et al.* High-pressure effects revisited for the cuprate superconductor family with highest critical temperature. *Nat. Commun.* 6:8990 doi: 10.1038/ncomms9990 (2015).

## Supplementary Material

Supplementary Information Supplementary Figures 1-3 and Supplementary Table 1.
