# Supplementary material for: High pressure effects revisited for the cuprate superconductor family with highest critical temperature
Source: Nat Commun. 2015 Dec 1;6:8990. doi: 10.1038/ncomms9990 (PMC4686855; doi:10.1038/ncomms9990)
Supplement: Supplementary Information — Supplementary Figures 1-3 and Supplementary Table 1. [file ncomms9990-s1.pdf]

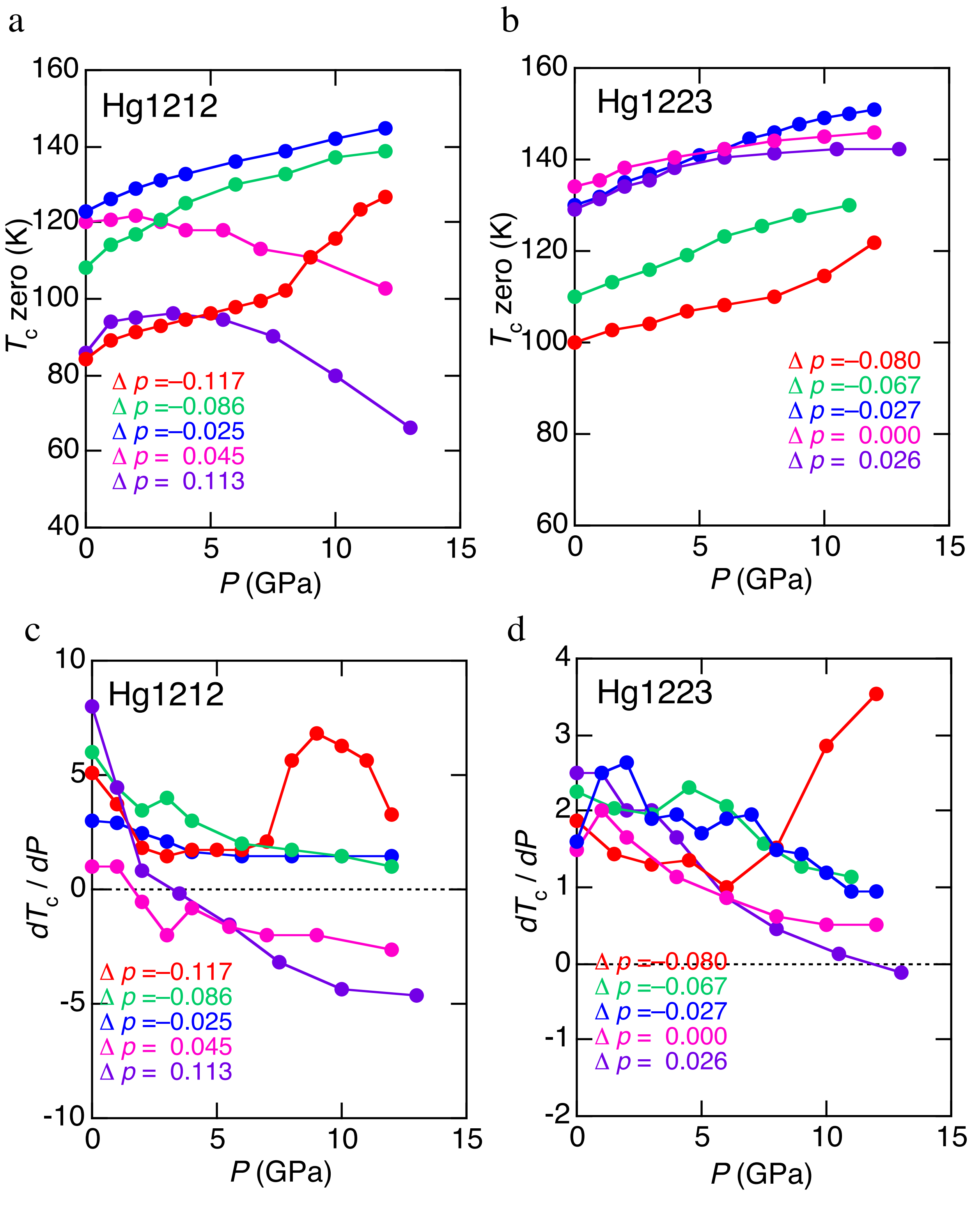

Supplementary Figure 1: Pressure dependence of T_c and dT_c/dP for Hg1212 and Hg1223 at high pressures over a wide doing range. (a, b) Pressure dependence of T_c for Hg1212 (**a**) and Hg1223(**b**), **(c, d)** Pressure dependence of dT_c/dP for Hg1212 (**c**) and Hg1223 (**d**).

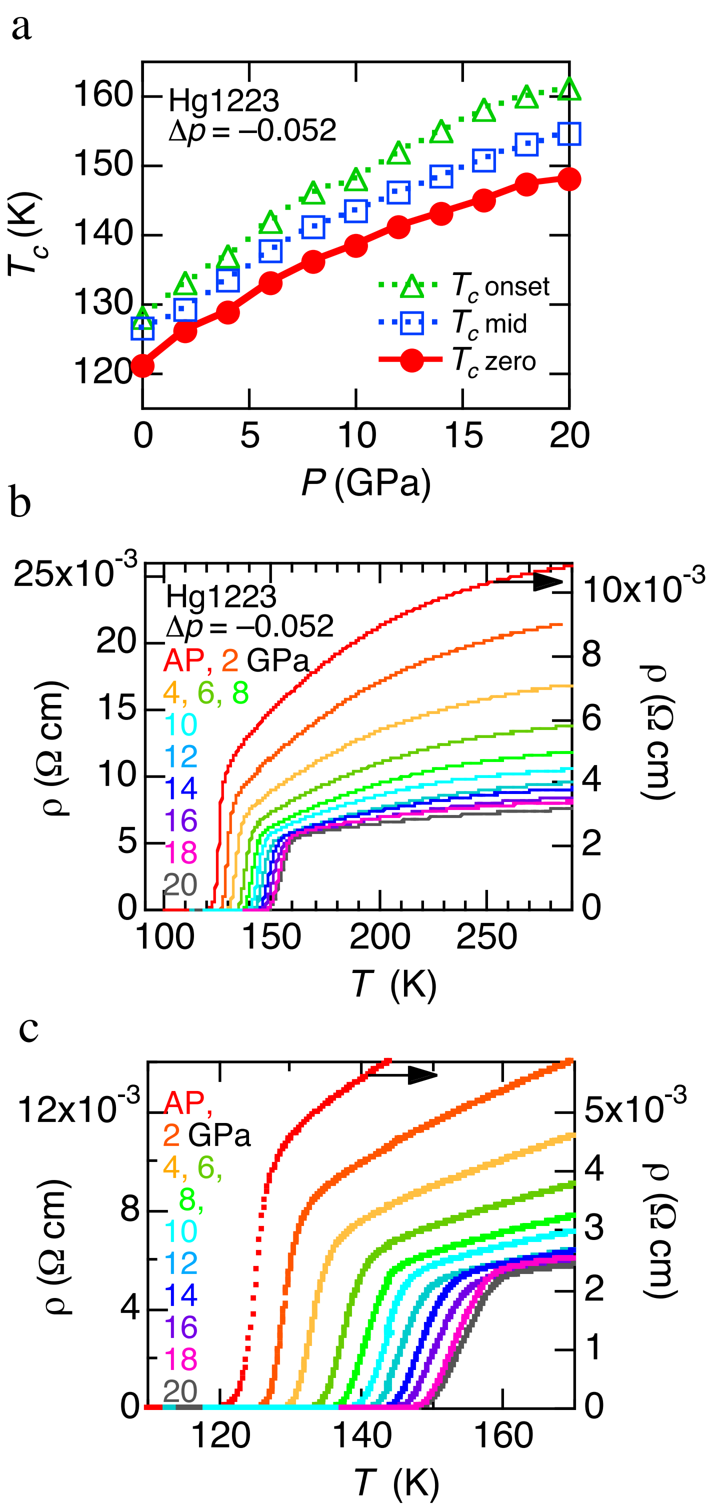

Supplementary Figure 2: Effects of pressure on superconductivity in underdoped Hg1223 up to 20 GPa. (**a**) Pressure (*P*) dependence of $T_{c\ zero}$ (zero-resistivity), $T_{c\ mid}$ (a peak position of dr/d*T* in superconducting transition), and $T_{c\ onset}$ (the resistivity to start to decrease toward zero, as defined by a rising edge in dr/d*T*) for underdoped Hg1223. (**b**) Temperature dependence of electrical resistivity (ρ–*T*) for underdoped Hg1223, Colour of curve corresponds to the text colour of pressures. **(c)** Zoom-up figure of **b**

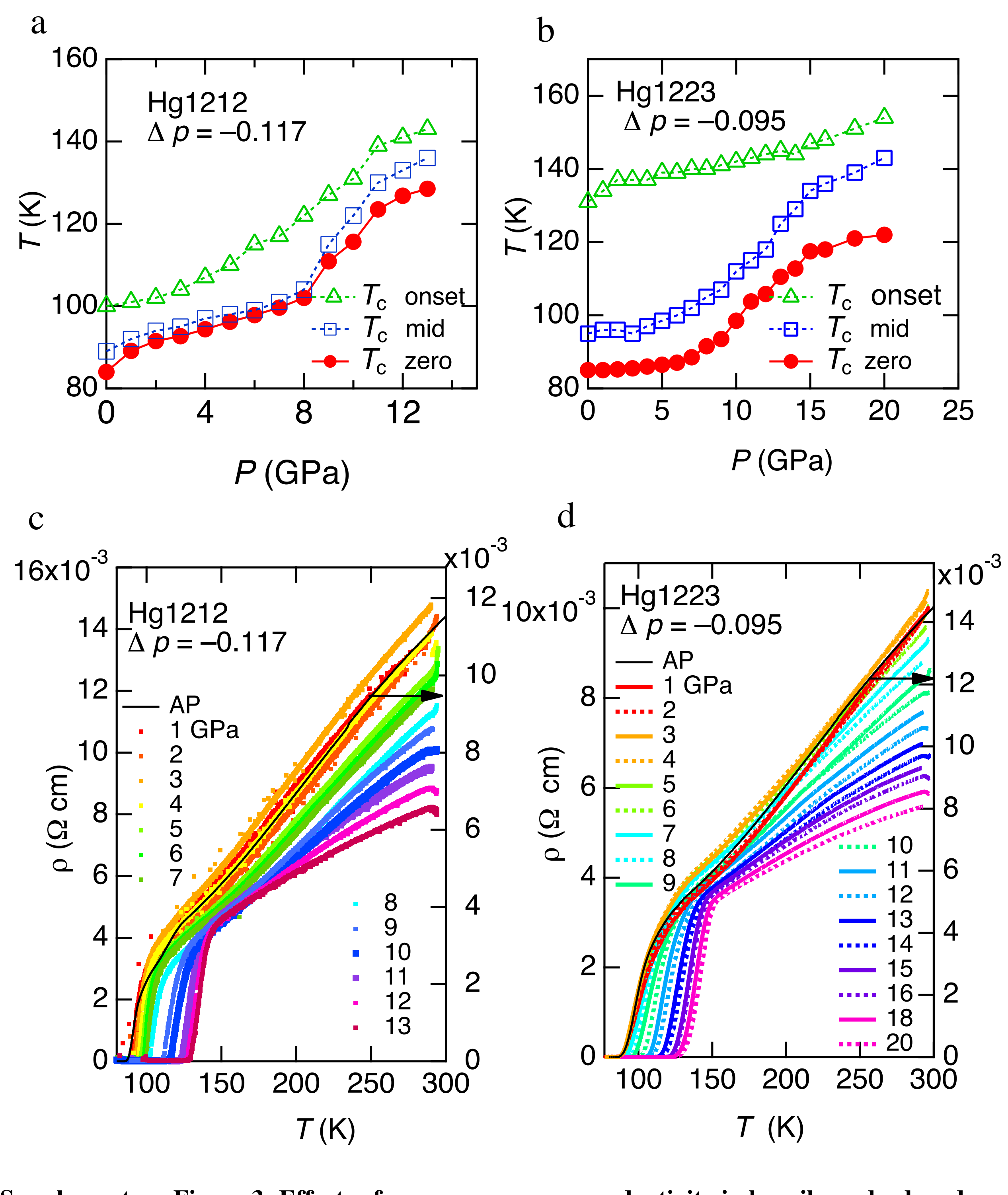

Supplementary Figure 3: Effects of pressure on superconductivity in heavily underdoped Hg1212 and Hg1223. (a, b) Pressure (*P*) dependence of $T_{c\ zero}$ (zero-resistivity), $T_{c\ mid}$ (a peak position of dr/d*T* in superconducting transition), and $T_{c\ onset}$ (the resistivity to start to decrease toward zero) for heavily underdoped Hg1212 **(a)** and **(b)**. **(c, d)** Temperature dependence of electrical resistivity (ρ–*T*) for heavily underdoped Hg1212 (c) and Hg1223 (d).

Supplementary Table1:

Synthesis and annealing condition of the samples. All samples sintered at 2 GPa using a cubic-anvil press.

(i) Hg1212

Figure	Δp	Starting material & sintering condition	Annealing condition
1, 2	–0.117	$HgO+BaO_2+CaO+CuO/Cu$, 835˚C, 10min	N_2, 500˚C, 1week
	–0.086	$HgO+BaO_2+CaO+CuO/Cu$, 835˚C, 10min	N_2, 400˚C, 1week
	–0.025	$HgO+BaO_2+CaO+CuO/Cu$, 835˚C, 10min	–
	0.045	HgO+ Ba-Ca-Cu-O, 850˚C, 30min	O_2, 360˚C, 1week
	0.113	HgO+ Ba-Ca-Cu-O, 850˚C, 30min	–
4	–0.062	$HgO+BaO_2+CaO+CuO/Cu$, 835˚C, 10min	N_2, 300˚C, 1week
S3	–0.117	$HgO+BaO_2+CaO+CuO/Cu$, 835˚C, 10min	N_2, 500˚C, 1week

(ii) Hg1223

Figure	Δp	Starting material & sintering condition	Annealing condition
1, 2	–0.080	HgO+ Ba-Ca-Cu-O(cooling 10˚C/min), 835˚C, 15min	N_2, 525˚C, 1week
	–0.067	HgO+ Ba-Ca-Cu-O(cooling 10˚C/min), 835˚C, 15min	N_2, 450˚C, 1week
	–0.027	HgO+ Ba-Ca-Cu-O(cooling 10˚C/min), 835˚C, 15min	–
	0.000	HgO+ Ba-Ca-Cu-O(cooling 10˚C/min), 805˚C, 20min	–
	0.026	HgO+ Ba-Ca-Cu-O(cooling 2˚C/min), 820˚C, 20min	–
4	–0.030	HgO+ Ba-Ca-Cu-O(cooling 5˚C/min), 820˚C, 20min	N_2, 340˚C, 1week
S2	–0.052	HgO+ Ba-Ca-Cu-O(cooling 5˚C/min), 820˚C, 20min	N_2, 300˚C, 1week
S3	–0.095	HgO+ Ba-Ca-Cu-O(cooling 5˚C/min), 820˚C, 20min	Vac., 550˚C, 72h
